# Toe-brachial index is beyond a peripheral issue in patients with type 2 diabetes

**DOI:** 10.1371/journal.pone.0253138

**Published:** 2021-06-15

**Authors:** Gisoo Darban Hosseini Amirkhiz, Mohammad Reza Babaei, Nahid Hashemi Madani, Mohammad Ebrahim Khamseh

**Affiliations:** 1 Research Center for Prevention of Cardiovascular Disease, Institute of Endocrinology and Metabolism, Iran University of Medical Sciences (IUMS), Tehran, Iran; 2 Department of Interventional Radiology, Firouzgar Hospital, Iran University of Medical Science (IUMS), Tehran, Iran; 3 Endocrine Research Center, Institute of Endocrinology and Metabolism, Iran University of Medical Sciences (IUMS), Tehran, Iran; Hospital Midt: Hospitalsenhed Midt, DENMARK

## Abstract

**Background:**

Atherosclerosis is the leading cause of death in patients with diabetes. We aimed to evaluate the association between ankle-brachial index (ABI) and toe-brachial index (TBI) with carotid intima-media thickness (CIMT) in patients with type 2 diabetes (T2DM).

**Methods:**

This cross sectional study included 296 patients with T2DM who met the American Diabetes Association criteria for the assessment of peripheral arterial atherosclerosis. The ABI ≤ 0.9 and TBI ≤ 0.7 were considered as abnormal values. Linear and logistic regression analyses were performed to evaluate the association between TBI/ABI and CIMT.

**Results:**

Right CIMT was significantly greater in the low TBI group (*p* = 0.03) while, left CIMT did not show a significant difference. Each 0.1-unit decrease in TBI value was independently associated with 0.017 mm increase in the right CIMT (β ± SE; -0.017 ± 0.005, *p* = 0.002) and with odds of the presence of increased CIMT [odds ratio and 95% confidence interval: 1.21 (1.02, 1.44)] after adjustment with all traditional risk factors. There was not any significant association between ABI and increased CIMT.

**Conclusions:**

Beyond a suitable tool for the diagnosis of peripheral artery disease in patients with T2DM, TBI can be applied for prediction of subclinical carotid atherosclerosis.

## Introduction

With the improvement in the life expectancy of human beings, early identification and timely treatment of chronic disease is of a significant importance. In the recent decades, atherosclerosis leading to the heart attack and stroke is the most common cause of death and morbidity worldwide [1]. Peripheral arterial disease (PAD) is atherosclerotic narrowing of arteries that supply organs other than heart and brain. It most commonly affects the lower extremity arteries. The growing body of knowledge suggesting the association between PAD and carotid artery atherosclerosis emphasizes the fact that atherosclerosis is a systemic disease, so that the presence of atherosclerosis in some area is a warning sign for that in the other regions [2]. Considering the growing number of patients with diabetes and the attributed double risk of developing cardiovascular and cerebrovascular events due to the atherosclerosis among these patients, screening tests for diagnosis of subclinical vascular problems in patients with type 2 diabetes (T2DM) seems to be essential [3–5].

Ankle-brachial index (ABI) is an inexpensive, easy-to-use, and widely available method for evaluating PAD and is correlated to the cardiovascular disease [6] Toe-brachial index (TBI) seems to provide a more accurate risk evaluation in patients with diabetes because medial calcification of the arteries frequently present in these patients and may lead to detection of a falsely normal ABI value underestimating the PAD [7, 8]. TBI is not affected by this phenomenon and may not only be a precise indicator of PAD but may also be a predictor of general arterial atherosclerosis in patients with diabetes.

Carotid intima-media thickness (CIMT) is an established marker for the diagnosis of subclinical atherosclerosis and a predictor for future cardiovascular events [9–11]. Vascular screening with ABI, TBI and CIMT in patients with T2DM gives invaluable information about the further need for medical or non-medical prophylactic interventions. Previous studies have shown the decreased ABI and increased CIMT are related to more severe coronary artery disease and increase the global atheroma burden and cardiovascular events [12, 13]. However, there is little research regarding the relation between TBI and CIMT. The aim of this study was to evaluate the association between ABI/ TBI and CIMT and also survey the ability of ABI/TBI to predict the increased CIMT in patients with T2DM.

## Materials and methods

### Participants

This is a cross-sectional study on the patients with T2DM referred to the foot clinic for routine checkups at Institute of Endocrinology and Metabolism, Iran University of Medical Sciences (IUMS) during 2018_2019. Ethical approval was obtained from the local ethics committee. All participants provided written informed consent before participation.

We included all patients with a history of decreased walking speed, claudication, or abnormal pedal pulse. Moreover, all asymptomatic patients aged more than 50 years or less than 50 years who have other PAD risk factors (e.g., smoking, hypertension, dyslipidemia, or duration of diabetes > 10 years) were also included in the study [14]. Patients with a history of severe congestive heart failure, lymphedema, bilateral mastectomy, cellulitis, vasospastic disorders (Raynaud’s phenomenon, Raynaud’s disease, livedo reticularis, and, acrocyanosis) thrombophlebitis, deep vein thrombosis (DVT) within the past 6 months, or presence of a wound preventing Doppler probe or ankle cuff placement, were excluded from the study. Patients with major amputation, morbid obesity, severe osteoarthritis, and those unable to walk were also excluded from the study.

### Clinical and laboratory data

Participants were asked to complete a brief questionnaire including demographic data, duration of diabetes, smoking status, medical history (hypertension (HTN), coronary artery disease (CAD), or stroke). In the same session measurement of blood pressure (BP), weight, and height as well as assessment for the presence of neuropathy were performed by an experienced nurse. Neurological exam was designed to identify loss of protective sensation (LOPS) [15]. The 10-g monofilament at four points (1st, 3rd, and 5th metatarsal heads and plantar surface of distal hallux) was tested on each foot. The 128 Hz tuning fork was applied over the tip of the great toe bilaterally to assess vibration perception. Ankle reflexes was tested while the patient is resting on the couch. Pinprick test was performed using a disposable pin which was applied just proximal to the toenail on the dorsal surface of the hallux. The presence of any abnormal test was considered as having LOPS.

Moreover, blood sample was obtained for the measurement of glycated hemoglobin (HbA1C), triglyceride (TG), total cholesterol (chol), low density lipoprotein (LDL), high density lipoprotein (HDL). All laboratory tests were performed at the institute’s laboratory.

### Vascular measures

The patients were asked to abstain from caffeine, cigarettes, and exercise at least two hours before the examination. After a 15-minute rest in the supine position, in a room with a constant temperature of 23_25°C, each participant underwent ABI and TBI measurement with Doppler ultrasound (DUS) (LifeDop 150; Summit Doppler, Wallach Surgical Devices, Turn-bull, Conn). All measurements were performed by an experienced nurse to avoid the interobserver discrepancies [16].

The toe pressure and the higher pressure between the arteries of posterior tibial (PT) and dorsalis pedis (DP) for each leg is divided into the higher blood pressure recorded from either arm to calculate the TBI and ABI for each lower extremity, respectively. For each patient the lower values of ABI and TBI of either side were used for the analysis. The ABI ≤ 0.9 and TBI ≤ 0.7 were considered abnormal [17]. The participants were categorized into two groups based on normal vs. abnormal ABI as well as TBI.

DUS of both common carotid arteries was performed by an experienced sonographer blinded to the other results with the MylabClassC color Doppler ultrasound diagnostic system (Esaote, Guangdong, China), using a 5–13 MHz vascular probe. In the supine position with a head rotated towards the opposite side, the CIMT was measured at the end of the diastole. The distance between the echogenic lines of intima-blood interface and outer adventitia-media junction in a plaque free area of the far wall considered as CIMT. All measurements was done in a longitudinal plane [18]. The CIMT greater than 0.82 and 0.85 mm for the right and left side were considered to be increased, respectively [19].

### Statistical analysis

All statistical analyses were conducted using IBM SPSS Statistics for Windows (Version 22.0 IBM Corp. Released 2013. Armonk, NY). Skewness and kurtosis of the continuous variables tested for the assumption of normality. Continuous variables were reported as mean ± standard deviation (SD) or median with interquartile range and the categorical variables were presented as a percentage. Between-group differences were examined using the χ2 test, independent sample t-test, or Mann-Whitney U test, as appropriate. TBI coefficient of correlation with age, gender, diabetes duration, Body Mass Index (BMI), CIMT, and laboratory measures were assessed. Linear and logistic regression analyzes were performed to evaluate the association between CIMT and TBI or ABI in the following 3 different models: the first unadjusted, the second adjusted for age and gender, and the third adjusted for age, gender, BMI, duration of diabetes, smoking status, HDL, LDL, and HbA1c. Models were checked for multi-collinearity and variance inflation factors (VIF) were below 2 for all variables.

Sample size with type 1 error of 0.05 and 95% confidence interval was calculated as 120 patients using the following equation. SD for CIMT in patients with T2DM assumed 0.2. (S1 File) [20].

## Results

Two hundred and ninety-six participants with T2DM met inclusion criteria. There were more women (n = 176) than men (n = 120). The mean age of the participants was 60.1±8.4 years and most of them (90%) were below 70 years old. The mean duration of diabetes was 13.4±8.4 years and only 20.6% had duration of fewer than 5 years. The mean BMI of the patients was 28.9±4.5 kg/m^2^. The percentage of current smokers was 6.4%. Most patients were asymptomatic and do not need any interventions. Only 13 participants needed endovascular intervention or revascularization. Asymptomatic patients had normal distribution for age and mean ± SD for this group was 59.6±8.1. Individuals were categorized by TBI and ABI status. About 41% of participants had low TBI (TBI≤0.7) while only 6% had low ABI (ABI≤0.9). Patients with lower ABI were more likely to be male, had a higher prevalence of neuropathy, CVD, smoking, and higher TG but lower HDL levels. There was no significant difference in age, duration of diabetes, BMI, systolic and diastolic blood pressure, HbA1c, total cholesterol, and LDL between the two ABI categories. The group with lower TBI had higher age, longer duration of diabetes, higher prevalence of CVD and neuropathy, and higher LDL level. There was no difference in gender, BMI, history of smoking, systolic and diastolic blood pressure, HbA1c, TG, total cholesterol, and HDL between the two categories of TBI. Right CIMT was significantly greater in the low TBI group compared to the high TBI counterparts (0.77± 0.16 vs 0.73±0.14, *p* = 0.03) and also in the low ABI group compared to high ABI counterparts (0.91± 0.19 vs 0.74±0.14, *p* = 0.001). However, there was no significant difference between left CIMT in different TBI/ABI categories. (Table 1).

**Table 1:** Characteristics of the study participants.

		TBI			ABI		
variable	total	≤0.7	>0.7	*p* value	≤0.9	>0.9	*p* value
N (%)	296	121(40.8)	175(59.1)		18(6.0)	278(93.9)	
Mean age(years)	60.1±8.4	61.4±9.2	59.3±7.8	0.04	62.5±11.3	59.9±8.1	0.36
Gender(%male)	40.5	44.6	37.7	0.23	72.2	38.4	0.005
Duration of diabetes (year)	13.4±8.4	14.9±9.2	12.3±7.6	0.01	14.3±8.7	13.3±8.4	0.61
BMI(kg/m^2^)	28.9±4.5	28.6±4.8	29.0±4.3	0.46	28.5±3.8	28.9±4.6	0.70
Current Smokers (%)	19 (6.4)	11(9.1)	8(4.6)	0.11	7(38.8)	12(4.3)	<0.001
Known CVD (%)	41(13.9)	23(19.0)	18(10.3)	0.03	7(38.9)	34(12.2)	<0.001
Neuropathy (%)	131(44.3)	65(53.7)	66(37.7)	0.006	14(77.8)	117(42.2)	0.003
SBP (mmHg)	125±14	127±15	124±14	0.12	137±28	130±19	0.15
DBP (mmHg)	80±9	81±8	79±10	0.10	81±11	77±10	0.13
HbA1c (%) (±SD)	7.7±1.6	7.8±0.1	7.7±0.1	0.83	7.9±1.5	7.7±1.6	0.65
TG (mg/dl) (±SD)	137.0±73.3	147.0±88.4	130.4±60.7	0.06	187.9±91.1	133.8±71.0	0.004
Total cholesterol (mg/dl) (±SD)	138.3±31.1	142.1±35.3	135.9±28.2	0.10	141.8±33.3	138.3±31.3	0.64
LDL (mg/dl) (±SD)	68.6±26.6	73.2±30.1	65.7±23.7	0.02	74.8±29.4	68.3±26.4	0.34
HDL (mg/dl) (±SD)	43.8±11.8	44.2±13.1	43.5±10.9	0.61	33.7±9.3	44.4±11.7	0.004
Right CIMT (mm) (±SD)	0.75±0.15	0.77±0.16	0.73±0.14	0.03	0.91±0.19	0.74±0.14	0.001
Left CIMT (mm) (±SD)	0.76±0.17	0.77±0.17	0.75±0.16	0.35	0.81±0.19	0.76±0.17	0.27

Continuous data are presented as means ± SD; categorical data are presented as percentage; N, number of patients; BMI, body mass index; Known CVD = myocardial infarction or stroke, PCI/bypass or angina; SBP, systolic blood pressure; DBP, diastolic blood pressure; TG, triglyceride; LDL, low density lipoprotein cholesterol; HDL, high density lipoprotein cholesterol; CIMT, carotid intima media thickness

**Fig 1:**
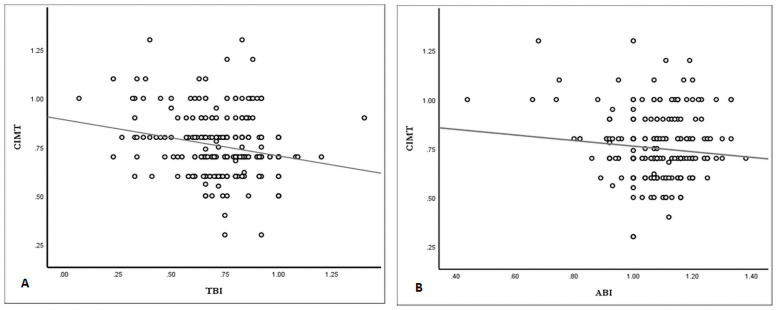
Graph illustrating correlation between right CIMT and TBI (A) and ABI (B). CIMT: carotid intima media thickness. TBI: toe brachial index. ABI: ankle brachial index.

TBI was correlated negatively with right CIMT (r = -0.21, *p*<0.001) but there was no significant correlation between TBI and left CIMT (r = -0.09, *p* = 0.08). Besides, ABI did not show any significant correlation with right CIMT (r = -0.10, *p* = 0.08) or left CIMT (r = 0.04, *p* = 0.49). (Fig 1).

The association between ABI/TBI and right CIMT as a continuous variable was studied applying linear regression analyzes. Each 0.1-unit decrease in TBI was associated with 0.017 mm increase in the right CIMT. This association was significant even after adjustment for age, gender, BMI, smoking status, HDL, LDL, and HbA1C (*p* = 0.002). On the contrary, there was no significant association between ABI and right CIMT in any of the models. (Table 2).

**Table 2:** Linear regression analysis of relation between TBI/ABI (0.1 unit) and CIMT (1mm).

	B ± SE	*p* value
TBI		
Model 1	- 0.019±0.005	<0.001
Model 2	-0.017±0.005	0.001
Model 3	-0.017±0.005	0.002
ABI		
Model 1	-0.014±0.008	0.08
Model 2	-0.015±0.008	0.061
Model 3	-0.014±0.008	0.081

Model 1: unadjusted; Model 2: adjusted for age and gender; Model 3: adjusted for age, gender, BMI, duration of diabetes, smoking status, HDL, LDL, HbA1c.

Logistic regression analyzes showed that each 0.1-unit decrease in TBI value was significantly associated with odds of increased right CIMT [odds ratio (OR) and 95% confidence interval (CI): 1.27 (1.08, 1.49) in model 1; 1.25 (1.06, 1.47) in model 2; and 1.21 (1.02, 1.44) in model 3]. There was not any significant association between ABI and increased right CIMT. (Table 3).

**Table 3:** Logistic regression analysis on relationship between ABI/TBI (0.1 unit) and high CIMT.

	Model 1	Model 2	Model
	OR (95% CI)	OR (95% CI)	OR (95% CI)
TBI	1.27 (1.08, 1.49)	1.25 (1.06, 1.47)	1.21 (1.02, 1.44)
ABI	1.19 (0.92, 1.53)	1.20 (0.93, 1.53)	1.15 (0.90, 1.48)

High CIMT is defined as CIMT≥0.82. Model 1: unadjusted; Model 2: adjusted for age and gender; Model 3: adjusted for age, gender, BMI, duration of diabetes, smoking status, HDL, LDL, HbA1c. OR, odds ratio; CI, confidence interval

## Discussion

In the current study, the relevance of ABI/TBI and ultrasound marker of carotid artery atherosclerosis, CIMT, was surveyed in the middle-aged patients with T2DM. There was a significant negative association between TBI and CIMT considering all potential risk factors. However, no relationship was observed between ABI and CIMT. Although decreased ABI is a well-known predictor of increased cardiovascular and all-cause mortality [21, 22], incompressible leg vessel due to the medial calcification in patients with diabetes makes ABI less sensitive in this population [23, 24]. Some previous studies have shown the superiority of TBI in predicting cardiovascular outcomes compared to ABI in the special populations [25]. Wang et al. [26] described a U-shaped relationship between ABI and CIMT obtained from the common carotid, internal carotid, and carotid bifurcation. Brasileiro et al. [27] observed a negative correlation between ABI and CIMT in their investigation but Wyman et al. [28], and Mitu et al. [29] found no association as ours. This discrepancy in the results might be due to the fact that the former study was done in the general population that lower proportion has diabetes. Arterial wall calcification in patients with diabetes interferes with the utility of the ABI [8]. Moreover, differences in age and ethnicity of the study populations as well as the methods and confounders are other explanations for these diverse results.

We found a significant negative correlation between TBI and CIMT both as a continuous and binary outcome. This result is consistent with the results of previous studies that had been conducted in a similar population of different ethnicities [20, 30, 31]. In our study, the right CIMT correlated better with TBI than did the left CIMT. Various studies have mentioned a significant difference between the right and left CIMT [32, 33]. A research conducted by Luo et al. indicated that risk factors affecting CIMT differed in the right and left sides. Right CIMT was more correlated with hemodynamic parameters while left CIMT was better correlated with biochemical and body composition changes including BMI, hip circumference, total cholesterol, LDL, and blood glucose [32]. Various effects of different risk factors on each CIMT seem to be the cause of the observed issue.

### Strength and limitations

To the best of our knowledge, this study is the first one that evaluated the association of TBI and CIMT in a population of Iranian people with T2DM. Moreover, all potential confounders have been taken into account. However, this is a single-center, cross-sectional study, and no casualty between TBI and CIMT could be concluded from it.

We found that the changes in CIMT values with decreasing TBI is too small to be measured with current methods. It highlights the need for further and more accurate techniques for CIMT evaluation.

## Conclusion

In summary, the present study demonstrated a significant negative association between TBI and right CIMT in patients with T2DM. The association was independent of all conventional risk factors. Thus, beyond a suitable tool for diagnosis of PAD in patients with diabetes, TBI can provide us with information on subclinical carotid atherosclerosis. This emphasized the necessity for more frequent use of TBI measurement, as a non-invasive, easily available, and low-cost tool, in diabetes clinics than previously recognized.

## Supporting information

S1 File Sample size formula. (DOCX)

S2 File Contribution of each element in linear regression analysis. (DOCX)

S3 File Contribution of each element in logistic regression. (DOCX)
