# Supplementary material for: Toe-brachial index is beyond a peripheral issue in patients with type 2 diabetes
Source: PLoS One. 2021 Jun 15;16(6):e0253138. doi: 10.1371/journal.pone.0253138 (PMC8205164; doi:10.1371/journal.pone.0253138)
Supplement: S2 File — (DOCX) [file pone.0253138.s002.docx]

S2 File. Contribution of each element in linear regression analysis. Linear regression analysis of relation between TBI/ABI (0.1 unit) and CIMT (1mm).

S2 Table 1. Linear regression analysis of relation between TBI/ABI (0.1 unit) and CIMT (1mm).

| Model | |  |  | *p* value | 95% CI | |
| --- | --- | --- | --- | --- | --- | --- |
|  |  | B | SE |  | Lower Bound | Upper Bound |
|  | TBI | -0.017 | 0.005 | 0.001 | -0.0273 | -0.0072 |
|  | Age | 0.0001 | 0.0001 | 0.277 | -0.0001 | 0.0003 |
|  | Gender (female vs. male) | -0.004 | 0.002 | 0.001 | -0.0082 | -0.0008 |
|  | ABI | -0.015 | 0.008 | 0.061 | -0.0312 | 0.0007 |
|  | Age | 0.0002 | 0.0001 | 0.152 | -0.0001 | 0.0004 |
|  | Gender (female vs. male) | -0.0049 | 0.0019 | 0.010 | -0.0087 | -0.012 |
|  |  |  |  |  |  |  |

Model 2: adjusted for age and gender; CI, confidence interval

S2 Table2. Linear regression analysis of relation between TBI/ABI (0.1 unit) and CIMT (1mm).

| Model | |  |  | *p* value | 95% CI | |
| --- | --- | --- | --- | --- | --- | --- |
|  |  | B | SE |  | Lower Bound | Upper Bound |
|  | TBI | -0.017 | 0.005 | 0.002 | -0.027 | -0.006 |
|  | Age | 0.0001 | 0.0001 | 0.378 | -0.0001 | 0.0003 |
|  | Gender (female vs. male) | -0.0025 | 0.0022 | 0.267 | -0.0068 | 0.0019 |
|  | Duration of DM | 0.0001 | 0.0001 | 0.454 | -0.0001 | 0.0003 |
|  | BMI | 0.0001 | 0.0002 | 0.570 | -0.0003 | 0.0006 |
|  | HbA1C | -0.0004 | 0.0006 | 0.563 | -0.0016 | 0.0008 |
|  | HDL | -0.0002 | 0.0001 | 0.007 | -0.0004 | -0.0001 |
|  | LDL | 0.0000 | 0.0000 | 0.429 | -0.0001 | 0.0000 |
|  | Smoking (smoker vs. non-smoker) | 0.0008 | 0.0041 | 0.851 | -0.0074 | 0.0089 |
|  | ABI | -0.0147 | 0.0084 | 0.081 | -0.0311 | 0.0018 |
|  | Age | 0.0002 | 0.0001 | 0.176 | -0.0001 | 0.0004 |
|  | Gender (female vs. male) | -0.0028 | 0.0023 | 0.211 | -0.0073 | 0.0016 |
|  | Duration of DM | 0.0001 | 0.0001 | 0.292 | -0.0001 | 0.0004 |
|  | BMI | 0.0002 | 0.0002 | 0.446 | -0.0003 | 0.0006 |
|  | HbA1C | -0.0003 | 0.0006 | 0.647 | -0.0015 | 0.0009 |
|  | HDL | -0.0002 | 0.0001 | 0.011 | -0.0004 | 0.0000 |
|  | LDL | 0.0000 | 0.0000 | 0.613 | -0.0001 | 0.0000 |
|  | Smoking (smoker vs. non-smoker) | 0.0024 | 0.0042 | 0.563 | -0.0058 | 0.0106 |

Model 3: adjusted for age, gender, BMI, duration of diabetes, smoking status, HDL, LDL, HbA1c; CI, confidence interval
