# Supplementary material for: Toe-brachial index is beyond a peripheral issue in patients with type 2 diabetes
Source: PLoS One. 2021 Jun 15;16(6):e0253138. doi: 10.1371/journal.pone.0253138 (PMC8205164; doi:10.1371/journal.pone.0253138)
Supplement: S3 File — (DOCX) [file pone.0253138.s003.docx]

S3 contribution of each element in logistic regression. Logistic Regression analysis on relationship between ABI/TBI (0.1 unit) and high CIMT.

S3 Table 1. Logistic Regression analysis on relationship between ABI/TBI (0.1 unit) and high CIMT.

|  | | *p* value | OR | 95% CI | |
| --- | --- | --- | --- | --- | --- |
|  |  |  |  | Lower | Upper |
|  | TBI | 0.007 | 1.25 | 1.06 | 1.47 |
|  | Age | 0.597 | 0.99 | 0.99 | 1.00 |
|  | Female | 0.010 | 0.92 | 0.86 | 0.98 |
|  | ABI | 0.143 | 1.20 | 0.93 | 1.53 |
|  | Age | 0.377 | 0.99 | 0.99 | 1.00 |
|  | Female | 0.006 | 0.91 | 0.86 | 0.97 |

High CIMT is defined as CIMT≥0.82. Model 2: adjusted for age and gender; OR, odds ratio; CI, confidence interval

S3 Table 2. Logistic Regression analysis on relationship between ABI/TBI (0.1 unit) and high CIMT.

|  | | *p* value | OR | 95% CI | |
| --- | --- | --- | --- | --- | --- |
|  |  |  |  | Lower | Upper |
|  | TBI | 0.028 | 1.21 | 1.02 | 1.44 |
|  | Age | 0.646 | 0.99 | 0.99 | 1.00 |
|  | Female | 0.104 | 0.94 | 0.87 | 1.01 |
|  | Duration of DM | 0.599 | 0.99 | 0.99 | 1.00 |
|  | BMI | 0.376 | 0.99 | 0.98 | 1.00 |
|  | HbA1C | 0.947 | 1.00 | 0.98 | 1.02 |
|  | HDL | 0.046 | 1.00 | 1.00 | 1.00 |
|  | LDL | 0.588 | 1.00 | 0.99 | 1.00 |
|  | Smoker | 0.923 | 0.99 | 0.87 | 1.12 |
|  | ABI | 0.249 | 1.15 | 0.90 | 1.48 |
|  | Age | 0.403 | 0.99 | 0.99 | 1.00 |
|  | Female | 0.092 | 0.94 | 0.87 | 1.01 |
|  | Duration of DM | 0.441 | 0.99 | 0.99 | 1.00 |
|  | BMI | 0.305 | 0.99 | 0.98 | 1.00 |
|  | HbA1C | 0.975 | 0.99 | 0.97 | 1.02 |
|  | HDL | 0.055 | 1.00 | 0.99 | 1.00 |
|  | LDL | 0.721 | 1.00 | 0.99 | 1.00 |
|  | Smoker | 0.814 | 1.01 | 0.89 | 1.14 |

High CIMT is defined as CIMT≥0.82. Model 3: adjusted for age, gender, BMI, duration of diabetes, smoking status, HDL, LDL, and HbA1c. OR, odds ratio; CI, confidence interval
