# Supplementary material for: Toe-brachial index is beyond a peripheral issue in patients with type 2 diabetes
Source: PLoS One. 2021 Jun 15;16(6):e0253138. doi: 10.1371/journal.pone.0253138 (PMC8205164; doi:10.1371/journal.pone.0253138)
Supplement: Supplementary file 3 [file pone.0253138.s001.docx]

$$n = \left(\frac{Z_{\frac{\propto}{2}} * \sigma}{\varepsilon} \right)^2$$
